# Acute compartment syndrome: Cause, diagnosis, and new viewpoint

**DOI:** 10.1097/MD.0000000000016260

**Published:** 2019-07-05

**Authors:** Jialiang Guo, Yingchao Yin, Lin Jin, Ruipeng Zhang, Zhiyong Hou, Yingze Zhang

**Affiliations:** a Department of Orthopaedic Surgery, The Third Hospital of Hebei Medical University; b Key Laboratory of Orthopaedic Biomechanics of Hebei Province, Shijiazhuang; c Orthopaedic Research Institution of Hebei Province, Hebei; d Chinese Academy of Engineering, Beijing, China.

**Keywords:** acute compartment syndrome, diagnosis, fasciotomy, mechanism

## Abstract

**Background:**

Acute compartment syndrome (ACS) is defined as a clinical entity originated from trauma or other conditions, and remains challenging to diagnose and treat effectively. The review was aim to present the controversy in diagnosing, treating ACS. It was found that there was no criterion about the ACS, and result unnecessary osteotomy. The presence of clinical assessment (5P) always means the necrosis of muscles and was the most serious or irreversible stage of ACS. Besides pressure methods, the threshold of pressure identifying ACS was also controversial.

**Methods:**

Immediate surgical fasciotomy was important to prevent severe suquelae of the ACS. However, there was still controversy about the right time that fasciotomy should be done to avoid irreversible ischemic changes. The most important thing to treat ACS was comprehension to the true injury mechanism, but a systemic classification about traumatic mechanism in most literature was not clear.

**Results:**

After observations to fracture patients with blister, we recommended that surgeons dealing with such emergencies should be vigilant, and the indication for fasciotomy should be strictly controlled following with injury mechanism especially for patients without severe soft tissue injury.

**Conclusion:**

For those crushing and soft tissue injuries, the current evidence based strategies for managing patients was useful, but for those fracture related injury, more examination was necessary to avoid overtreatment especially for those patients with blister observed. In facing patients, medical history, injured mechanism should be paid special attention, and rigorous classification about traumatic etiology was the key for the treatment of these patients.

## Abstract 2

Supplemental Digital Content is available in the text

## 1 Introduction

Acute compartment syndrome (ACS) is defined as a clinical entity originated from trauma or other conditions that cause bleeding, edema, or that compromises perfusion in limbs. It was described around 130 years ago, and remains challenging to diagnose and treat effectively.^[1]^ After a decrease in a compartment volume or/and an increase in the contents of a compartment, ACS occurs when pressure increases within a confined closed fascial space causing subsequently reduced blood flow and tissue perfusion that may lead to ischemic pain, possible soft-tissue damage, and limb-threatening. With a character of increased intracompartmental pressure, it is a surgical emergency and commonly occurs in the lower leg, forearm, thigh, foot, gluteal region, hand, and abdomen. The incidence rate of ACS was reported 30.4% especially in shaft and proximal regions of tibia. The higher incidence of ACS in proximal tibia is directly related to high energy trauma causing comminuted fractures, especially those affecting the entity of medial tibial plateau and increased with the fracture line extended laterally.^[2–5]^

To prevent serious complications induced by ACS, fasciotomy should be done before irreversible tissue necrosis occurs, thus there is a strong clinic bias toward doing fasciotomy empirically or prophylactically in patients who are considered to be at high risk and/or who have concerning clinical findings. It is generally recommended that conducting a prophylactic fasciotomy, which may be unnecessary, is better than do it too late, or ignoring a true ACS, given the potential systemic risks (rhabdomyolysis and myonecrosis) and functional loss associated with untreated ACS (Supplemental Figure 1). However, there were controversies regarding the diagnosis (method and pressure threshold), treatment (the time of fasciotomy), and injury mechanism of ACS. The aim of this review was to present the uncertainties in treating ACS and propose new solutions.

## 2 Diagnosis varieties

The patients’ photographs were collected from our institutional database, retrospectively, and all the methods were performed in accordance with the relevant guidelines and regulations of the Third Hospital of Hebei Medical University. It was approved by the committee of the Third Hospital of Hebei Medical University.

The diagnosis of compartment syndrome is always controversial and is based on clinical assessment and pressure measurement in compartment. Compartment syndrome clinical hallmarks have been defined as the 5Ps: pain out of proportion, pallor, paresthesias, paralysis, and pulselessness. Someone considered 5Ps as the established diagnostic procedures monitoring ACS, but it is an unreliable determinant of the presence of the syndrome, and many of clinical symptoms and signs also occur in patients without ACS (reamed nailing to tibia fractures with a sign of common or deep peroneal nerve), perhaps due to direct tissue injury 10 to 18. It is also not useful in patients with a decreased level of consciousness, unconscious, or insensate who are unable to provide feedback. In fact, the presence of these signs always means the necrosis of muscles and was the most serious or irreversible stage of ACS. Furthermore, these signs are more often signs of arterial ischemia than ACS, and be elicited only in the fully alert patient.^[6–8]^

The surgeons concern mainly about clinical signs of ACS such as worsening pain that is out of proportion and increasing analgesic requirements, or anxiety, agitation. However, the subjective symptoms rely heavily on clinical expertise, are impossible to standardize, and meanwhile the early changes of ischemia in compartment was the increased pressure.^[9]^ Ulmer found that clinical examination had poor sensitivity and a high negative predictive value, which means that it is better in excluding than confirming ACS.^[7]^ Therefore, subjective clinical assessments of compartments are unreliable even judged by experienced clinicians, the results was that unnecessary liberal fasciotomy which expose patients to an increased risk of complications from wound infection was more than we can image. In the case that the clinical diagnosis is equivocal, measurement of intracompartmental tissue pressure might be helpful because of the fact that the pressure changes pull ahead the clinical symptoms and signs.^[10]^ Through pressure monitoring, it reduces both the delay to fasciotomy and the development of sequelae, but the researches that identified the pressure as the measuring criteria was only 11.7%.^[11]^ An investigation reveals that only about half of the hospitals in Germany have the technical equipment to do this despite most surgeons’ agreeing that pressure measurement is the standard method.

There are many methods of monitoring pressure, and the equipment was different from each other. Invasive choices of monitoring pressure include the Whitesides needle manometer, a slit or wick catheter. The deficiency of these devices was that it was easily to be blocked by lest muscle and blood clots. The STIC Monitor (Stryker Orthopaedics, Mahwah, NJ) is a portable monitor that uses a side port needle, a disposable syringe of saline flush, and a digital read out manometer to allow for simple measurement of compartment pressure. The measurement will be effected by position, measuring location and tissue in tip of needle. Through knee cadaveric specimens, large calculated interobserver technical variations, and errors in the measurement of compartment pressures, he found that even with proper technique, 40% of the measurements were >5 mm Hg from the actual pressure. Therefore, it was misleading to make the choice depending on pressure monitor; many patients may be wrongly diagnosed as ACS. Some researches considered that the pressure monitor by needle was technically wrong in clinic. Nudel et al found that ACS is not uniform about pressure distribution in the compartment.^[6]^ The pressure adjacent to the damaged artery is substantially higher than a critical value, indicating the need for fasciotomy. However, at the same time, the pressure in regions far from the bleeding artery is substantially lower than the threshold value even after 2 hours from injury. Intracompartmental pressure was highest and should be measured within 5 cm of the site of fracture.^[12,13]^ However, the standard whether one should obtain pressures near the fracture, or measure further away (outside the zone of injury) to obtain a more representative pressure to the majority of the compartment was not established.

Besides pressure methods, the threshold of pressure identifying ACS was also controversial. It was identified a compartment pressure of 30 mm Hg was resistant to infusion of fluid and recommended as indication of surgical fasciotomy.^[14]^ Levels ranging from 30 to 50 mm Hg are also proposed as critical level to diagnose ACS.^[14–16]^ However, the value was proved to be inaccurate, and no correlation was found between high compartment pressure and clinical outcomes in patients treated primarily in plaster cast.^[17]^ More and more researches demonstrated that pressures more than 30 mm Hg can also be tolerated without sequel, and proposed that instead of an absolute threshold, the difference between diastolic pressure and compartment pressure was accepted as indication for fasciotomy (diastolic pressure minus compartment pressure <30 mm Hg).^[18–21]^ However, along with pressure measurement, it is important that the diagnosis of ACS should take into account time factors, and single pressure measurements alone reflect instantaneous blood perfusion. The tissue and blood pressure was dynamic equilibrium, and pressure in different compartment was various from each other. Bussell et al reported that pressure in the anterior compartment was higher compared to all the other compartments within the healthy and fractured leg in children.^[22]^ McQueen et al monitored pressure for 2 hours and demonstrated excellent sensitivity (94%) and specificity for ACS after tibia fractures.^[23]^ On the contrary, there was also researches reported that continuous compartment pressure monitoring was not advocated in alert patients.^[24]^ Diagnosis based on pressure measurements (diastolic pressure minus compartment pressure <30 mm Hg) alone is also reported unreliable. Ho found high compartment pressures are frequently seen in patients with tibial shaft fractures, but in most cases, it does not equate to the presence of compartment syndrome, and unnecessary fasciotomy should be avoided.^[25]^ Prayson et al followed blood pressure and compartment pressure in 19 patients with lower extremity fractures, and also did not found compartment syndrome with the perfusion pressure threshold (<30 mm Hg).^[26]^ Janzing and Broos reported that 45.4% of patients would have undergone fasciotomies using Δ*P* under 30 mm Hg, leading to a number of unnecessary operations and complications.^[27]^

The overtreatment based on intracompartmental pressure measurements alone was still existed in a sizeable number of patients, and not all hospitals have the technical equipment to do this. In a war, the clinical practice guidelines for treating patients do not support the use of pressure measurements due to time limited, the early and liberal use of prophylactic fasciotomies was advocated. Although early, fasciotomy has reduced the incidence of ACS among soldiers, it comes at a cost in terms of associated morbidities such as infection, nerve injury, and sensory, following surgery for delayed wound closure with high cost. The incidence of complications associated with fasciotomy has been reported to be as high as 87% following battlefield trauma.^[28]^ In earthquake, fasciotomy was always carried out without pressure measurement.^[29]^

Above all, when the clinical observations are inconclusive, pressure measurement can be helpful to confirm or exclude the diagnosis, not as a screening tool for those with an increased risk of developing compartment syndrome. However, there was no standard criterion even when clinical indication and pressure threshold combined that enable a successful prediction of the need for surgery treatment. And after considering the confusion about ACS, we begin to doubt the accuracy of ACS, and the existence of ACS.

## 3 The time of fasciotomy and results

As we mentioned earlier, in traditional view, immediate surgical fasciotomy was important to prevent severe suquelae of the ACS. However, there was still controversy about the right time that fasciotomy should be done to avoid irreversible ischemic changes. The ischemic necrosis of muscle can be observed as early as 3 hours, 5% may be injured after 4 hours, and become permanent in 8 hours.^[30,31]^ Labbe et al reported that when the ischemia was 3, 4, and 5 hours, the necrosis of leg muscles was up to 20%, 30%, and 90%, respectively.^[32]^ On the contrary, Sheridan and Matsen found that 68% of 22 patients treated within 12 hours recovered normal lower extremity function compared to only 8% treated after 12 hours.^[33]^ There was not only damage to muscles but also to the associated nerves traversing the compartment if compartment syndrome is not diagnosed and treated early. It was reported that if compartmental release was performed within 4 hours, the nerve conduction velocity returned to normal regardless of the amount of pressure or time of the pressure applied. However, nerve conduction velocity will not return to normal if the release was performed after 12 hours. The late diagnosis may result in the possibility of irreversible nerve, muscle damage, amputation, and even death.

Despite there is obvious evidence that delay in treatment leads to poorer outcomes, it is difficult to determine the exact time of performance for fasciotomy. For delayed compartment syndrome in adults, someone propose that routine fasciotomy should be conducted to prevent more morbidity and complication.^[34]^ In contrast, others suggested that 8 hours of ischemia can result in permanent myonecrosis, so traditional fasciotomies should be restricted as fasciotomies can no longer reverse the nerve damage and muscle necrosis, and the procedure may potentially lead to wound problems and infection. Reis and Michaelson suggested that the skin serves an important role as a barrier to infection, and patients with closed crush injuries treated by late fasciotomy (≥24 hours) had worse outcomes than those treated nonoperatively.^[35]^ Finkelstein et al also reported that infection after delayed fasciotomy has greater morbidity than the muscle contractures occurred from myonecrosis.^[36]^ Ritenour et al reported that wounded soldiers that had delayed fasciotomies, had higher rates of muscle excision, amputation, and mortality.^[37]^ Therefore, prophylactic fasciotomy can also induce major complications, and the risk–benefit ratio should be weighed heavily.

However, functional outcomes were different and controversial in different aged patients. Livingston et al reported the time from symptom onset or initial injury to ACS diagnosis in nonfracture pediatric patients to be over 48 hours.^[38]^ After a system review, Lin and Balch Samora reported that pediatric patients could still achieve functional recovery in 24 hours.^[34]^ Kanj et al found that although ACS of the upper extremity in children is often associated with a long delay between injury and fasciotomy, most children still achieve excellent outcomes.^[39]^ Above all, increased intracompartmental pressure can be tolerated for longer time compared with adults before tissue necrosis becomes irreversible. There was more time allowed to diagnose pediatric ACS after identifying the exact mechanism of injury.

## 4 Injury mechanism and fasciotomy results

The consequences of missed diagnosis are severe for both patients and surgeons. It is important to be cognizant that there is no universal pressure measurement that serves as the threshold for fasciotomy. The main etiologies of ACS was traumatic injuries such as fracture and crush-type injury, while other injuries such as limb ischemia (ischemia-reperfusion injury after revascularization), tourniquet, tight splint, shock trousers, drug injection, or snake bites could also induce ACS.^[40]^ Furthermore, the most important thing to treat ACS was comprehension to the true injury mechanism, but a systemic classification about traumatic mechanism in most literature was not clear. Patients in most articles can be classified into soft-tissue injury related, vascular injury related, fracture related according to the injured anatomical structure.^[41]^

Soft-tissue injury can be considered as crush syndrome. Patients with crush syndrome are induced by continuous prolonged pressure on muscle tissues, and character with massively swollen limbs, shock, myoglobinuria, and renal failure. Although fasciotomy is considered as the gold standard for ACS, but the role of fasciotomy in the treatment of crush syndrome is still controversial. The most debate was that in one hand, early fasciotomy prevents further muscle damage, and there were researches recommend fasciotomy for patients with crush syndrome.^[29,42,43]^ On the other hand, the fasciotomy convert a closed wound into an open one which increases the rate of infection. Despite high or increasing intracompartmental pressure, conservative treatment was advocated unless open wound existed or the limb circulation is decreased.^[35,44–46]^ For those patients with the necrotic muscle, Huang et al suggested that fasciotomy offers no benefit but increases the rate of infection and amputation.^[47]^

The ACS was always found to be mixed with the concept of crushing syndrome and Volkmann contracture. Crush syndrome is a medical condition that can be caused by a “crush injury,” and skeletal muscle becomes damaged under the heavy weight. The injury mechanism includes earthquakes, traffic accident, and war conflict and so on. The saved entrapped victims without immediate medical treatment caused the damage to the structure of compartment and lead muscle necrotic or damaged, and induce acute kidney injury that requires rapid and special treatment. The early debridement (not fasciotomy) should only be considered in patients with crushing injuries. Vascular injury associated with orthopedic trauma is also a potentially limb- and life-threatening, if the diagnosis of an associated vascular injury is missed or delayed, Volkmann contracture presented in late status (Table 1).

**Table 1:**
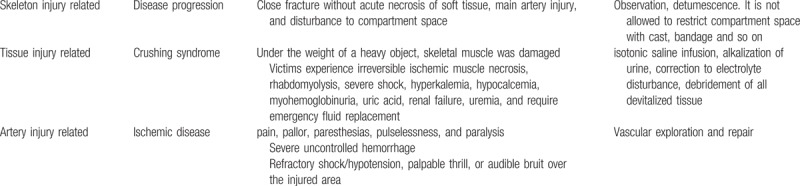
Different treatment strategy for different mechanism injuries.

Reperfusion was one reason reported for the development of ACS, and different from complete ischemia, ACS causes myonecrosis in the face of patent vessels 68. Furthermore, there are also debates regarding the role of early fasciotomy in vascular-injury-related patients. On one hand, the proponents argue that the morbility and amputation rates were unacceptable if the fasciotomy was delayed, so early fasciotomy has been cited as a major factor contributing to limb salvage and preservation of function, especially in vascular trauma patients. It was recommended as routine procedure along with vascular repair especially when popliteal arterial injury is associated with concomitant venous injury.^[48]^ On the other hand, infection associated with open fasciotomy incisions has been concerned. Although the reperfusion in patients with vascular injury was existed, but with an intact structure, nerve and function may recover later with some extents. Anecdotal indicated that infections after fasciotomy may result in leg amputation.^[49,50]^ Rollins et al also considered that the concept of fasciotomy for revascularization syndrome is rarely indicated.^[51]^ Abouezzi et al concluded that the presence of combined vascular injury especially the ones above the knee did not necessitate routine fasciotomy.^[52]^ Furthermore, the fasciotomy was also harmful to the function of limb in long term even there was no evidence of venous obstruction or reflux.^[53]^ In patients with persistent absent pulses or other sign of vascular injury, immediate vascular surgery consultation should be obtained.

Although some authors reported that tibial fracture with segmental tibia fracture, medial knee fracture-dislocation had a high rate of ACS, the patients diagnosed intraoperatively by objective compartment measurements may have never developed clinical ACS, and the mechanism classification should be reviewed carefully especially for severe tibial plateau fractures.^[5]^ Fracture is considered as a major contributing factor for ACS (approximately 75%).^[19,54–56]^ In patients with fracture, the structure in compartment was actually not disturbed which is different from crushing injury. In patients with crushing, cellular death or cell membrane lysis releases osmotically active cellular contents into the interstitial space, causing further accumulation of fluid and further increase in intracompartment pressure. In fracture-related patients, the cellular death or cell membrane lysis may be not common. The obvious differences of pressure increase among fracture, and crushing injury was that the former can be released though some mechanism surrounding intact fascia. If the patients was classified as fracture related, it stands a good chance that blister on the skin which reflects a release mechanism of pressure was observed, then the microcirculation of the tissues in that compartment is recovered soon or later (Supplemental Figures 2–8).

Fracture blisters were reported as a relatively uncommon complication of high-energy fractures (2.9%), but most tibial fracture can be observed with clear fluid or blood filled blister as early as 6 hours postfracture. It was considered that the factor in development of blister is injury to the dermal–epidermal junction resulting from high shear in the skin. The retention of some degree of epidermal cells in the clear-filled blisters contributed to a faster reepithelializaiton compared with blood-filled blister. However, the theory cannot explain the blister remote from the fracture deformity.^[57]^ Halawi also found that the blister which may induce infection can be observed after primary total knee arthroplasty, and the etiology of blister was multifactorial.^[58]^ The structure of skin was actually intact and without dermal–epidermal injuries excluding incision after arthroplasty, and we propose that the blister was more than just skin injury, and might be an efficient mechanism to release pressure. To be accordant with our hypothesis, Varela et al also reported that it is one of the mechanisms to relieve abnormally high pressure in compartment.^[59]^ Although there was no enough laboratory results to support our hypothesis, it can still be considered as an absolute mechanism to release pressure after tibial or ankle fracture from clinical observation, and the necessary of fasciotomy to fracture-related patients should be deliberate. Following experiment will be conducted soon to verify the hypothesis.

## 5 Conclusion

The ACS is considered as an orthopedic emergency which can lead to limb and life-threatening outcome if there is delay in diagnosis and treatment. Surgeons that involved in dealing with such emergencies should be vigilant, and the indication for fasciotomy should be strictly controlled following with injury mechanism especially for patients without severe soft-tissue injury. For those crushing and soft-tissue injuries, the current evidence-based strategies for managing patients were useful, but for those fracture-related injury, more examination was necessary to avoid overtreatment especially for those patients with blister observed. In facing patients’ medical history, injured mechanism should be paid special attention, and rigorous classification about traumatic etiology was the key for the treatment of these patients.

## Author contributions

Zhiyong Hou designed the study. Jialiang and Yingchao Yin conducted the procedure and collected the data of the study. Jialiang Guo, Lin Jin and Ruipeng Zhang drafted the manuscript. Zhiyong Hou and Yingze Zhang revised and approved the final manuscript.

**Conceptualization:** Zhiyong Hou.

**Data curation:** Yingchao Yin.

**Supervision:** Zhiyong Hou, Yingze Zhang.

**Validation:** Zhiyong Hou.

**Visualization:** Zhiyong Hou.

**Writing – original draft:** Jialiang Guo, Lin Jin, Ruipeng Zhang.

**Writing – review & editing:** Yingze Zhang.

## Supplementary Material

Supplemental Digital Content

## References

[1] von Volkmann R Ischaemic muscle paralyses and contractures. 1881. Clin Orthop Relat Res 2007;456:20–1. 17496749 10.1097/BLO.0b013e318032561f

[2] Acklin YP Potocnik P Sommer C Compartment syndrome in dislocation and non-dislocation type proximal tibia fractures: analysis of 356 consecutive cases. Arch Orthop Trauma Surg 2012;132:227–31. 21997400 10.1007/s00402-011-1408-0

[3] Ziran BH Becher SJ Radiographic predictors of compartment syndrome in tibial plateau fractures. J Orthop Trauma 2013;27:612–5. 23454859 10.1097/BOT.0b013e31828e25b6

[4] Chang YH Tu YK Yeh WL Tibial plateau fracture with compartment syndrome: a complication of higher incidence in Taiwan. Chang Gung Med J 2000;23:149–55. 15641218

[5] Stark E Stucken C Trainer G Compartment syndrome in Schatzker type VI plateau fractures and medial condylar fracture-dislocations treated with temporary external fixation. J Orthop Trauma 2009;23:502–6. 19633459 10.1097/BOT.0b013e3181a18235

[6] Nudel I Dorfmann L deBotton G The compartment syndrome: is the intra-compartment pressure a reliable indicator for early diagnosis? Math Med Biol 2017;34:547–58. 27756790 10.1093/imammb/dqw016

[7] Ulmer T The clinical diagnosis of compartment syndrome of the lower leg: are clinical findings predictive of the disorder? J Orthop Trauma 2002;16:572–7. 12352566 10.1097/00005131-200209000-00006

[8] Shuler FD Dietz MJ Physicians’ ability to manually detect isolated elevations in leg intracompartmental pressure. J Bone Joint Surg Am 2010;92:361–7. 20124063 10.2106/JBJS.I.00411

[9] Bae DS Kadiyala RK Waters PM Acute compartment syndrome in children: contemporary diagnosis, treatment, and outcome. J Pediatr Orthop 2001;21:680–8. 11521042

[10] McQueen MM Christie J Court-Brown CM Compartment pressures after intramedullary nailing of the tibia. J Bone Joint Surg Br 1990;72:395–7. 2341435 10.1302/0301-620X.72B3.2341435

[11] O’Toole RV Whitney A Merchant N Variation in diagnosis of compartment syndrome by surgeons treating tibial shaft fractures. J Trauma 2009;67:735–41. 19820579 10.1097/TA.0b013e3181a74613

[12] Heckman MM Whitesides TE Jr Grewe SR Compartment pressure in association with closed tibial fractures. The relationship between tissue pressure, compartment, and the distance from the site of the fracture. J Bone Joint Surg Am 1994;76:1285–92. 8077257 10.2106/00004623-199409000-00002

[13] Matava MJ Whitesides TE Jr Seiler JG 3rd Determination of the compartment pressure threshold of muscle ischemia in a canine model. J Trauma 1994;37:50–8. 8028059 10.1097/00005373-199407000-00010

[14] Mubarak SJ Owen CA Hargens AR Acute compartment syndromes: diagnosis and treatment with the aid of the wick catheter. J Bone Joint Surg Am 1978;60:1091–5. 721856

[15] Allen MJ Stirling AJ Crawshaw CV Intracompartmental pressure monitoring of leg injuries. An aid to management. J Bone Joint Surg Br 1985;67:53–7. 3968144 10.1302/0301-620X.67B1.3968144

[16] Mubarak SJ Hargens AR Acute compartment syndromes. Surg Clin N Am 1983;63:539–65. 6346542 10.1016/s0039-6109(16)43030-6

[17] Triffitt PD Konig D Harper WM Compartment pressures after closed tibial shaft fracture. Their relation to functional outcome. J Bone Joint Surg Br 1992;74:195–8. 1544950 10.1302/0301-620X.74B2.1544950

[18] Shadgan B Menon M O’Brien PJ Diagnostic techniques in acute compartment syndrome of the leg. J Orthop Trauma 2008;22:581–7. 18758292 10.1097/BOT.0b013e318183136d

[19] Elliott KG Johnstone AJ Diagnosing acute compartment syndrome. J Bone Joint Surg Br 2003;85:625–32. 12892179

[20] White TO Howell GE Will EM Elevated intramuscular compartment pressures do not influence outcome after tibial fracture. J Trauma 2003;55:1133–8. 14676660 10.1097/01.TA.0000100822.13119.AD

[21] McQueen MM Court-Brown CM Compartment monitoring in tibial fractures. The pressure threshold for decompression. J Bone Joint Surg Br 1996;78:99–104. 8898137

[22] Bussell HR Aufdenblatten CA Subotic U Compartment pressures in children with normal and fractured lower extremities. Eur J Trauma Emerg Surg 2019;4: doi: 10.1007/s00068-019-01082-9. 10.1007/s00068-019-01082-9 30715553

[23] McQueen MM Christie J Court-Brown CM Acute compartment syndrome in tibial diaphyseal fractures. J Bone Joint Surg Br 1996;78:95–8. 8898136

[24] Harris IA Kadir A Donald G Continuous compartment pressure monitoring for tibia fractures: does it influence outcome? J Trauma 2006;60:1330–5. 16766979 10.1097/01.ta.0000196001.03681.c3

[25] Lor KKH Yeoh NCS Wong KP Raised compartment pressures are frequently observed with tibial shaft fractures despite the absence of compartment syndrome: a prospective cohort study. J Orthop Surg (Hong Kong) 2017;25:2309499017717362 doi:10.1177/2309499017717362. 28664769 10.1177/2309499017717362

[26] Prayson MJ Chen JL Hampers D Baseline compartment pressure measurements in isolated lower extremity fractures without clinical compartment syndrome. J Trauma 2006;60:1037–40. 16688067 10.1097/01.ta.0000215444.05928.2f

[27] Janzing HM Broos PL Routine monitoring of compartment pressure in patients with tibial fractures: beware of overtreatment!. Injury 2001;32:415–21. 11382429 10.1016/s0020-1383(01)00005-5

[28] Masini BD Racusin AW Wenke JC Acute compartment syndrome of the thigh in combat casualties. J Surg Orthop Adv 2013;22:42–9. 23449054 10.3113/jsoa.2013.0042

[29] Oda J Tanaka H Yoshioka T Analysis of 372 patients with Crush syndrome caused by the Hanshin-Awaji earthquake. J Trauma 1997;42:470–5. 9095115 10.1097/00005373-199703000-00015

[30] Whitesides TE Heckman MM Acute compartment syndrome: update on diagnosis and treatment. J Am Acad Orthop Surg 1996;4:209–18. 10795056 10.5435/00124635-199607000-00005

[31] Heckman MM Whitesides TE Jr Grewe SR Histologic determination of the ischemic threshold of muscle in the canine compartment syndrome model. J Orthop Trauma 1993;7:199–210. 8326422 10.1097/00005131-199306000-00001

[32] Labbe R Lindsay T Walker PM The extent and distribution of skeletal muscle necrosis after graded periods of complete ischemia. J Vasc Surg 1987;6:152–7. 3039184 10.1067/mva.1987.avs0060152

[33] Sheridan GW Matsen FA 3rd Fasciotomy in the treatment of the acute compartment syndrome. J Bone Joint Surg Am 1976;58:112–5. 1249096

[34] Lin JS Balch Samora J Pediatric acute compartment syndrome: a systematic review and meta-analysis. J Pediatr Orthop B 2019;25: doi: 10.1097/bpb.0000000000000593. 10.1097/BPB.0000000000000593 30688754

[35] Reis ND Michaelson M Crush injury to the lower limbs. Treatment of the local injury. J Bone Joint Surg Am 1986;68:414–8. 3949835

[36] Finkelstein JA Hunter GA Hu RW Lower limb compartment syndrome: course after delayed fasciotomy. J Trauma 1996;40:342–4. 8601846 10.1097/00005373-199603000-00002

[37] Ritenour AE Dorlac WC Fang R Complications after fasciotomy revision and delayed compartment release in combat patients. J Trauma 2008;64:S153–61. 18376159 10.1097/TA.0b013e3181607750

[38] Livingston K Glotzbecker M Miller PE Pediatric nonfracture acute compartment syndrome: a review of 39 cases. J Pediatr Orthop 2016;36:685–90. 26019026 10.1097/BPO.0000000000000526

[39] Kanj WW Gunderson MA Carrigan RB Acute compartment syndrome of the upper extremity in children: diagnosis, management, and outcomes. J Child Orthop 2013;7:225–33. 24432081 10.1007/s11832-013-0492-9 PMC3672459

[40] Du W Hu X Shen Y Surgical management of acute compartment syndrome and sequential complications. BMC Musculoskelet Disord 2019;20:98. 30832634 10.1186/s12891-019-2476-5 PMC6399970

[41] Stella M Santolini E Sanguineti F Aetiology of trauma-related acute compartment syndrome of the leg: a systematic review. Injury 2019;2: doi: 10.1016/j.injury.2019.01.047. 10.1016/j.injury.2019.01.047 30772051

[42] Mubarak S Owen CA Compartmental syndrome and its relation to the crush syndrome: a spectrum of disease. A review of 11 cases of prolonged limb compression. Clin Orthop Relat Res 1975;81–9. 10.1097/00003086-197511000-00012 1192679

[43] Sheng ZY Medical support in the Tangshan earthquake: a review of the management of mass casualties and certain major injuries. J Trauma 1987;27:1130–5. 3312621

[44] Storgaard M Rasmussen K Ebskov B Traumatic rhabdomyolysis. Physiopathology and treatment [in Danish]. Ugeskr Laeger 1998;160:987–90. 9477744

[45] Michaelson M Crush injury and crush syndrome. World J Surg 1992;16:899–903. 1462627 10.1007/BF02066989

[46] Matsuoka T Yoshioka T Tanaka H Long-term physical outcome of patients who suffered crush syndrome after the 1995 Hanshin-Awaji earthquake: prognostic indicators in retrospect. J Trauma 2002;52:33–9. 11791049 10.1097/00005373-200201000-00008

[47] Huang KC Lee TS Lin YM Clinical features and outcome of crush syndrome caused by the Chi-Chi earthquake. J Formos Med Assoc 2002;101:249–56. 12101860

[48] Yeon J Jung YW Yang SS Lower limb compartment syndrome by reperfusion injury after treatment of arterial thrombosis post-laparoscopic radical hysterectomy and pelvic lymph node dissection for cervical cancer. Obstet Gynecol Sci 2017;60:223–6. 28344966 10.5468/ogs.2017.60.2.223 PMC5364107

[49] Feliciano DV Cruse PA Spjut-Patrinely V Fasciotomy after trauma to the extremities. Am J Surg 1988;156:533–6. 3202268 10.1016/s0002-9610(88)80547-6

[50] Lim LT Michuda MS Flanigan DP Popliteal artery trauma. 31 consecutive cases without amputation. Arch Surg 1980;115:1307–13. 7436723 10.1001/archsurg.1980.01380110045007

[51] Rollins DL Bernhard VM Towne JB Fasciotomy: an appraisal of controversial issues. Arch Surg 1981;116:1474–81. 7305661 10.1001/archsurg.1981.01380230088014

[52] Abouezzi Z Nassoura Z Ivatury RR A critical reappraisal of indications for fasciotomy after extremity vascular trauma. Arch Surg 1998;133:547–51. 9605919 10.1001/archsurg.133.5.547

[53] Bermudez K Knudson MM Morabito D Fasciotomy, chronic venous insufficiency, and the calf muscle pump. Arch Surg 1998;133:1356–61. 9865656 10.1001/archsurg.133.12.1356

[54] Olson SA Glasgow RR Acute compartment syndrome in lower extremity musculoskeletal trauma. J Am Acad Orthop Surg 2005;13:436–44. 16272268 10.5435/00124635-200511000-00003

[55] McQueen MM Gaston P Court-Brown CM Acute compartment syndrome. Who is at risk? J Bone Joint Surg Br 2000;82:200–3. 10755426

[56] Patel RV Haddad FS Compartment syndromes. Br J Hosp Med 2005;66:583–6. 10.12968/hmed.2005.66.10.19898 16255266

[57] Giordano CP Scott D Koval KJ Fracture blister formation: a laboratory study. J Trauma 1995;38:907–9. 7602633 10.1097/00005373-199506000-00014

[58] Halawi MJ Fracture blisters after primary total knee arthroplasty. Am J Orthop 2015;44:E291–3. 26251947

[59] Varela CD Vaughan TK Carr JB Fracture blisters: clinical and pathological aspects. J Orthop Trauma 1993;7:417–27. 8229378 10.1097/00005131-199310000-00004

