# Supplementary material for: Acute compartment syndrome: Cause, diagnosis, and new viewpoint
Source: Medicine (Baltimore). 2019 Jul 5;98(27):e16260. doi: 10.1097/MD.0000000000016260 (PMC6635163; doi:10.1097/MD.0000000000016260)

**Supplementary figure**

For fracture related injuries, our experience in treating these injurie was that the patients with fracture related (excluding vascular injury especially in femur fracture, knee dislocation, and severe tibial plateau fracture) suspected as ACS do not need fasciotomy, and the ACS was not actually existed. We called it law of self-releasing that means the increased pressure in compartment can be released with some unknown mechanisms. Fasciotomy to these patients was overtreatment, just like a patient who wants to suicide through clutching at throat by her hand, but when a threshold of force is reached she will become syncope and the clutching force is self-released. The surgeon did not realize it and broke her arms to save her.

**Supplemental Figures 1**


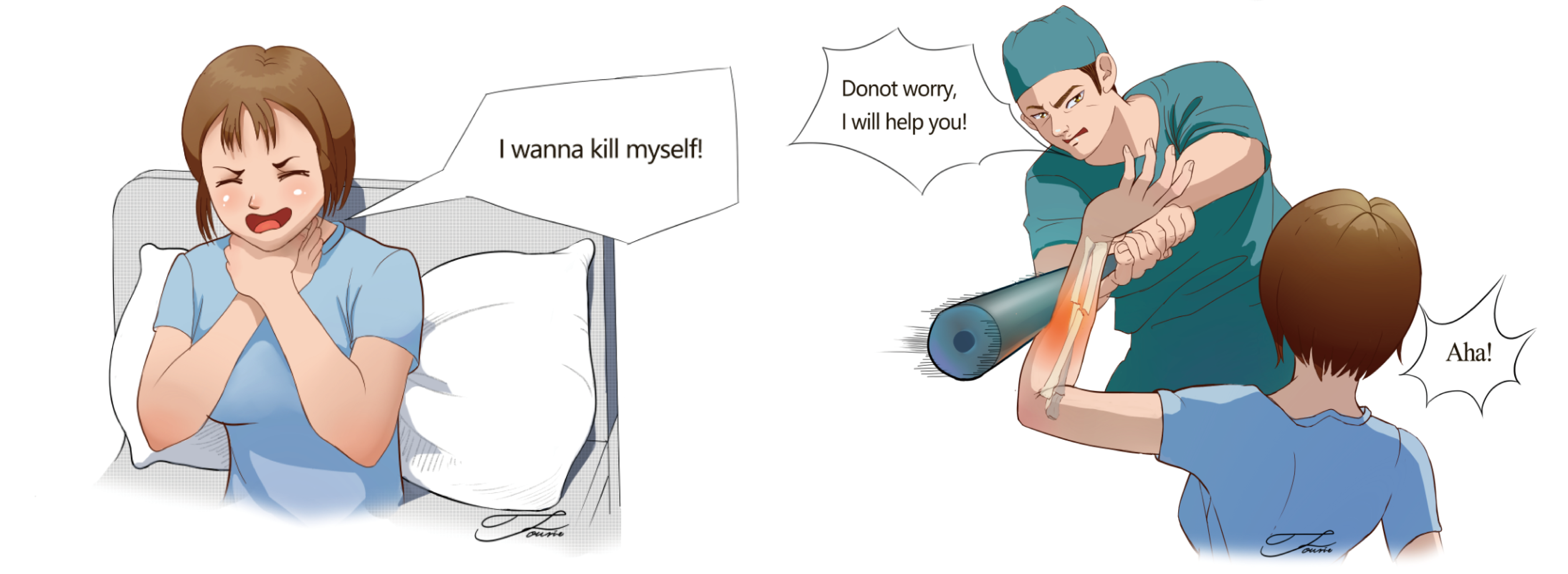


**Supplemental Figures 2**


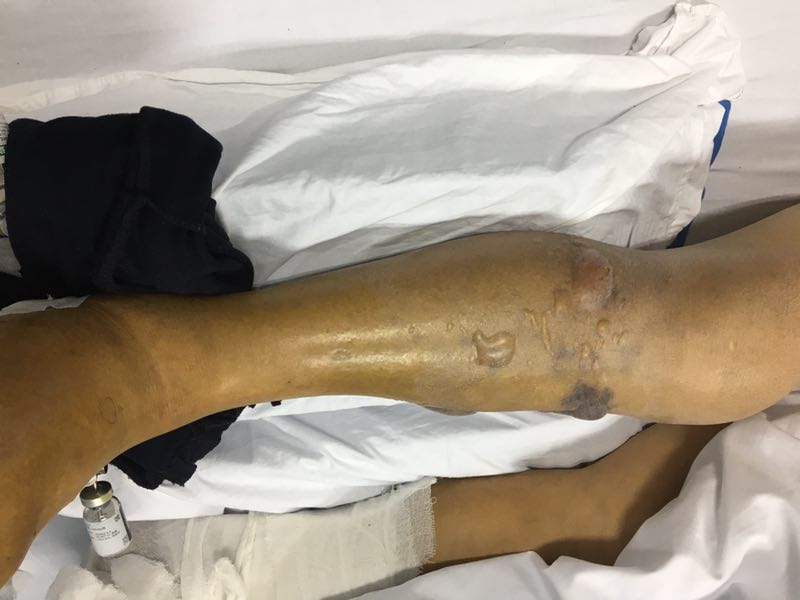


**Supplemental Figures 3**

**
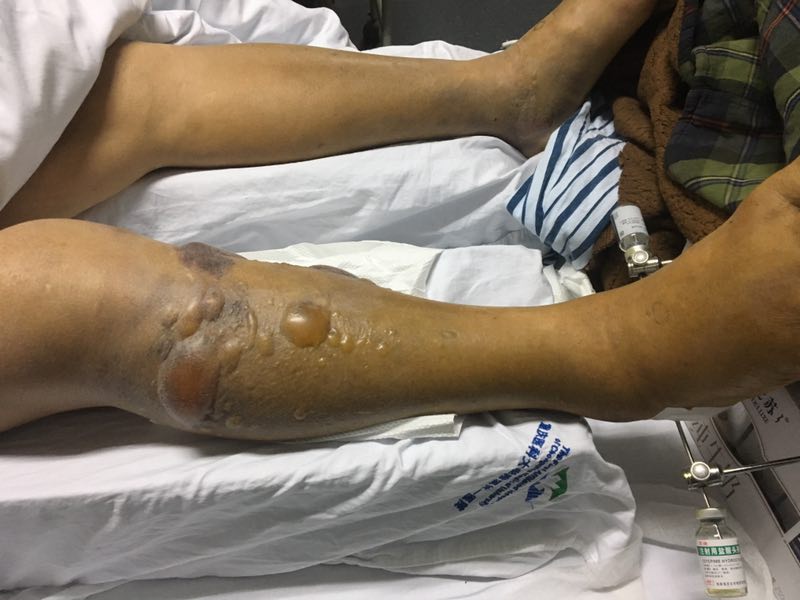
**

**Supplemental Figures 4**

**
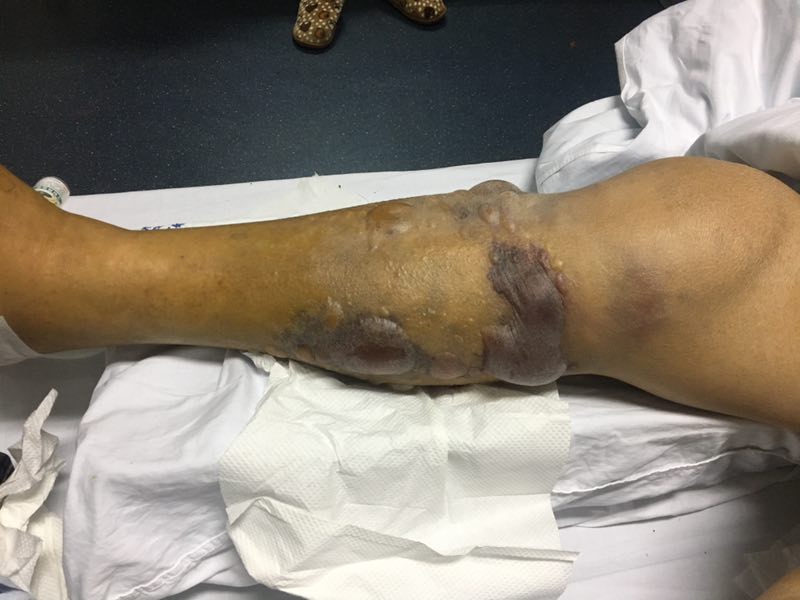
**

**Supplemental Figures 5**

**
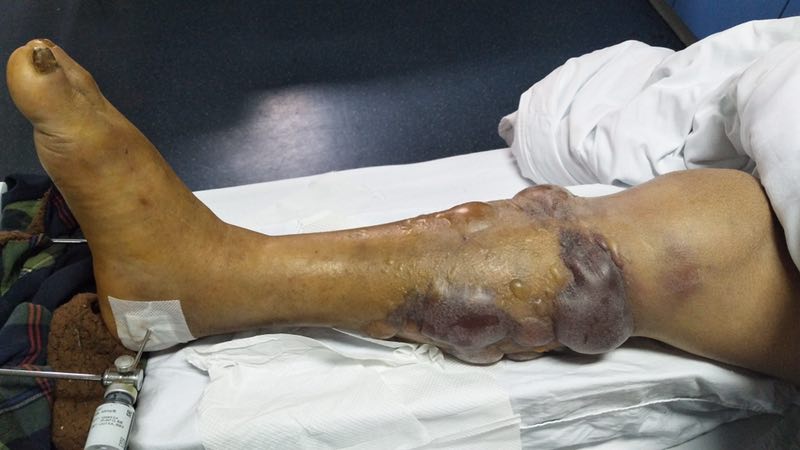
**

**Supplemental Figures 6**

**
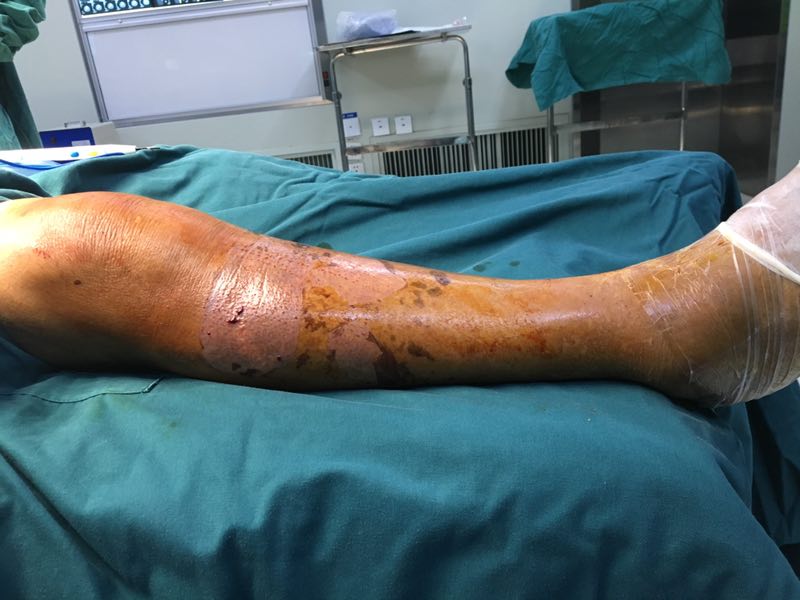
**

**Supplemental Figures 7**


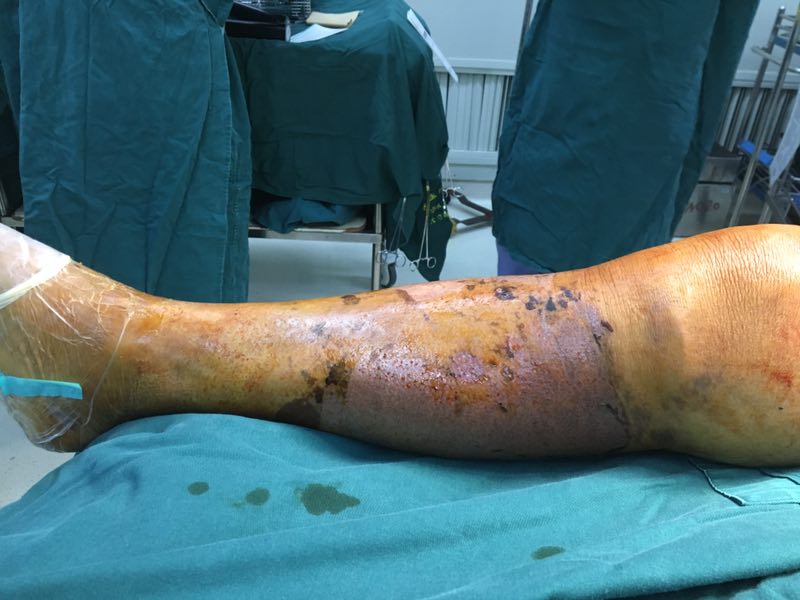


**Supplemental Figures 8**


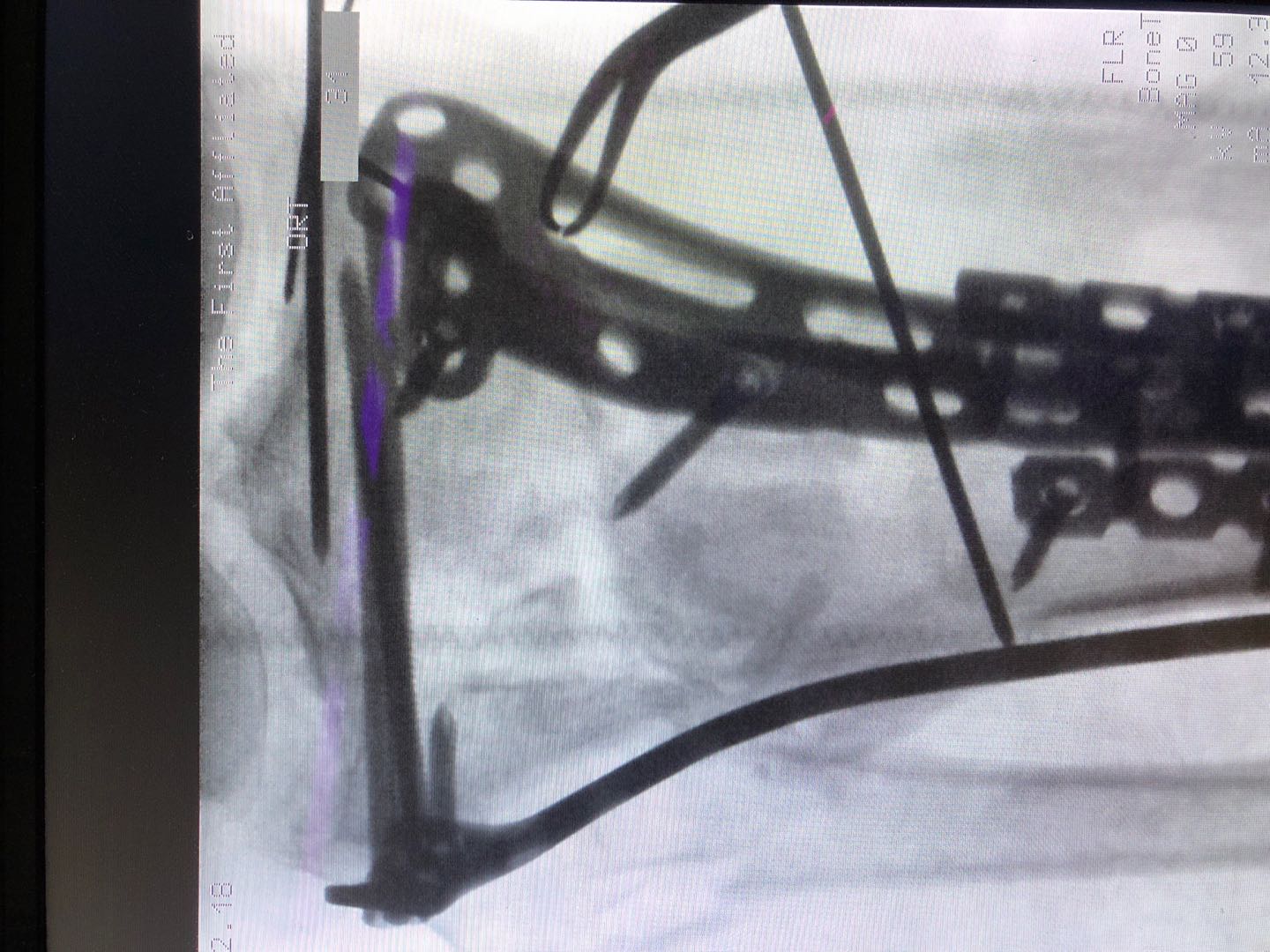

Supplement: Supplemental Digital Content [file medi-98-e16260-s001.doc]
